# A Rat Model of Radiation Vasculitis for the Study of Mesenchymal Stem Cell-Based Therapy

**DOI:** 10.1155/2019/3727635

**Published:** 2019-03-06

**Authors:** Jian Zhang, Xuan Tao, Mingyang Sun, Rongchao Ying, Wenjie Su, Wei Wei, Xiaohu Meng

**Affiliations:** ^1^Department of Gastroenterological Surgery, Affiliated Hangzhou First People's Hospital, Zhejiang University School of Medicine, Hangzhou, China; ^2^Division of General Surgery, The Second Affiliated Hospital of Nanjing Medical University, Nanjing, China; ^3^Division of General Surgery, Hangzhou First People's Hospital Affiliated to Nanjing Medical University, Hangzhou, China; ^4^Department of Vascular Surgery, Affiliated Hangzhou First People's Hospital, Zhejiang University School of Medicine, Hangzhou, China

## Abstract

Radiation vasculitis is one of the most common detrimental effects of radiotherapy for malignant tumors. This is developed at the vasculature of adjacent organs. Animal experiments have showed that transplantation of mesenchymal stem cells (MSCs) restores vascular function after irradiation. But the population of MSCs being engrafted into irradiated vessels is too low in the conventional models to make assessment of therapeutic effect difficult. This is presumably because circulating MSCs are dispersed in adjacent tissues being irradiated simultaneously. Based on the assumption, a rat model, namely, RT (radiation) plus TX (transplantation), was established to promote MSC homing by sequestering irradiated vessels. In this model, a 1.5 cm long segment of rat abdominal aorta was irradiated by 160kV X-ray at a single dose of 35Gy before being procured and grafted to the healthy counterpart. F344 inbred rats served as both donors and recipients to exclude the possibility of immune rejection. A lead shield was used to confine X-ray delivery to a 3 cm×3 cm square-shaped field covering central abdominal region. The abdominal viscera especially small bowel and colon were protected from irradiation by being pushed off the central abdominal cavity. Typical radiation-induced vasculopathy was present on the 90^th^ day after irradiation. The recruitment of intravenously injected MSCs to irradiated aorta was significantly improved by using the RT-plus-TX model as compared to the model with irradiation only. Generally, the RT-plus-Tx model promotes MSC recruitment to irradiated aorta by separating irradiated vascular segment from adjacent tissue. Thus, the model is preferred in the study of MSC-based therapy for radiation vasculitis when the evaluation of MSC homing is demanding.

## 1. Introduction

Radiotherapy is used to treat a variety of cancers, but the therapeutic index of radiotherapy is still limited by normal tissue injury in organs at risk. Vascular injury is the major cause of late radiation morbidity. The patients with head and neck tumors have a higher risk of developing dementia and cognitive dysfunction after radiotherapy [1]. Despite direct ionizing radiation injury to neurons and glial cells, brain blood circulation disorder resulting from vascular injury is an essential contributory factor [2, 3]. Small bowel is very vulnerable to irradiation. Chronic radiation enteritis is initiated as early as two months after patients received abdominal/pelvic irradiation, progressing throughout the rest of their life [4]. This is characterized by progressive obliterative arteritis with submucosal fibrosis [4]. Typically, the irradiated vessels develop slowly toward vascular fibrosis with luminal stenosis, excessive extracellular matrix deposition in the media and adventitia, intimal hyperplasia, and thrombus formation [3, 5]. The process has been replicated by rat models, in which radiation-induced vasculopathy is present three to six months after irradiation [6, 7]. Many potential therapeutic strategies have been investigated in these models prior to their clinical use.

Stem cell therapy holds great promise for radiation-induced vascular injury. Mesenchymal stem cells (MSC) are multipotent stromal cells that can differentiate into a variety of mature cell types. Moreover, MSCs themselves secrete a broad spectrum of trophic factors that serve to structure regenerative microenvironments [8]. MSCs were first separated from bone marrow and later found in other mesenchyme tissues. Their ease of isolation, manipulation, and potential use for tissue regeneration are specifically what have made them so attractive [9]. A number of animal studies have demonstrated that MSCs restore vascular function by both intravascular injection and seeding of vascular graft [6, 8, 10–12]. Nevertheless, the number of circulating MSCs that exactly reach irradiated vessels after intravascular injection is fairly low [8]. This is a practical problem in MSC-based therapy for radiation injury and other diseases as well [8, 13]. In a sense, the scarcity of MSCs in irradiated vessels renders assessment of therapeutic effect somewhat difficult, given that MSC recruitment is a prerequisite for effective cell-based therapy [8, 14, 15]. One possible explanation for the phenomenon is that transplanted MSCs are dispersed in adjacent tissue which is inevitably irradiated but constitutes a large compartment of MSC recruitment. In that case, the effort to localize radiation exposure probably enables MSCs to aggregate in irradiated vessels. The assumption is supported by the previous investigation of quantitative and spatial distribution of infused MSCs after local irradiation. Total body irradiation stimulated MSCs homed at a very low level to various tissues of the whole body, while additional local irradiation resulted in significant MSC engraftment in the exposed area [16]. Inspired by the findings, this study introduced a rat model, namely, RT (radiation) plus TX (transplantation), in which irradiated vessels were sequestered from adjacent tissue to promote MSC local recruitment.

## 2. Material and Methods

### 2.1. Animal

**Figure 1:**
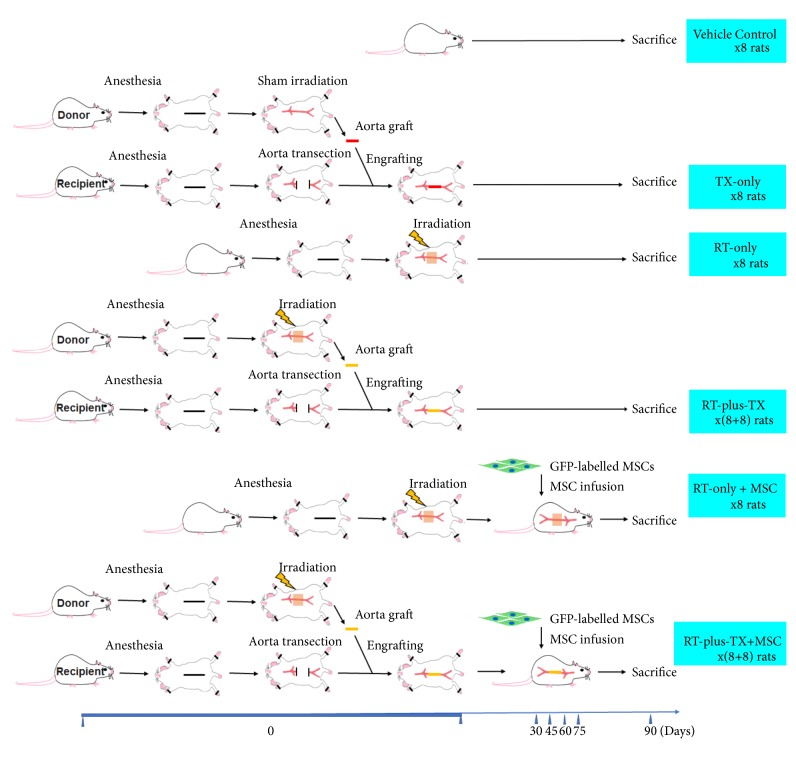
Animal groups and treatment protocols. Sixty-four female F344 rats were allocated to six groups. The RT-plus-TX and RT-plus-TX + MSC group each had eight pairs of rats, and the other groups each had eight rats. Aorta irradiation was conducted in four groups: RT-only, RT-only + MSC, RT-plus-TX, and RT-plus-TX + MSC groups. The TX-only and vehicle groups were not irradiated and served as negative control. After irradiation, the aortas from RT-plus-TX and RT-plus-TX + MSC groups were transplanted to healthy rats. The RT-only and RT-only + MSC groups served as negative control for aorta transplantation. The mesenchymal stem cells were infused to RT-only + MSC and RT-plus-TX + MSC groups starting from thirty days after irradiation for four times with the interval of fifteen days. All rats were sacrificed on the ninetieth day after irradiation, and the aortas were procured for histology and biomedical analysis.

Female F344 rats at 12 weeks of age (with average body weight of 200 g) were purchased from Vital River Laboratory Animal Technology (Beijing, China). The rats were housed at Laboratory Animal Center of Nanjing Medical University according to the guidelines of the National Institutes of Health Guide for the Care and Use of Laboratory Animals (NIH Publications No. 8023, revised 1978). During the following experiments, the rats were maintained in a specific pathogen-free grade barrier facility with a 12hr/12hr light-dark cycle, temperature at 18-22°C, and relative humidity at 40-60%. The rats were fed on standard pelleted food and water. Totally sixty-four rats were divided into six groups according to the treatment protocols (Figure 1). All animal procedures were approved by Committee of Animal Experiment Ethnics at Nanjing Medical University (Nanjing, China).

### 2.2. Abdominal Irradiation

**Figure 2:**
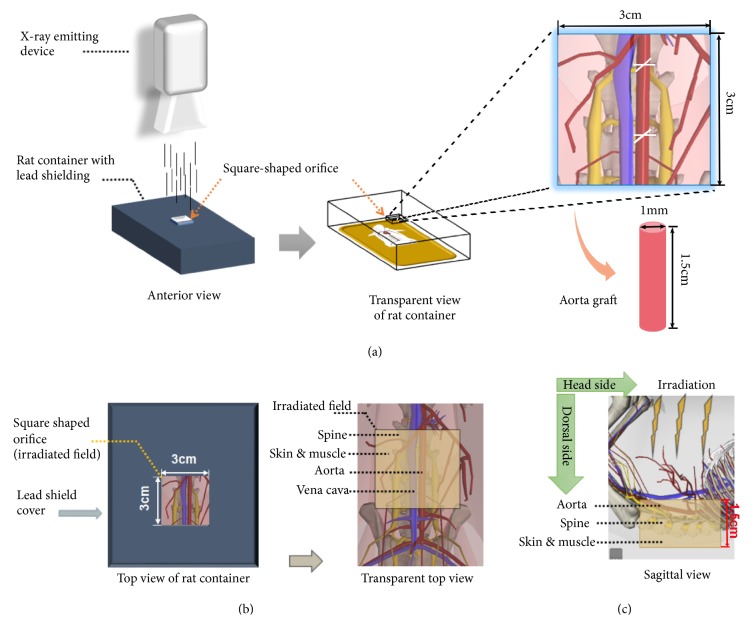
Schematic diagram of ionizing radiation device, lead shield, and irradiated field. (a) The rats were fixed in the sterilized container after anesthesia. The abdominal cavity was opened to expose the abdominal aorta in the center of abdomen. The X-ray beam was limited to 3cm×3cm square-shaped area of the central abdomen while another part of body was protected by a customized lead shield. After irradiation, a 1.5 cm long aorta graft was procured for transplantation. (b) The aorta together with the adjacent tissues was exposed to radiation through a square-shaped orifice on the top of lead shielding container. The irradiated adjacent tissues consisted of vena cava, spine, skin, and muscle in posterior abdominal wall. (c) The thickness of rat posterior abdominal wall was estimated at 1.5cm on average.

The rat was anesthetized by intraperitoneal injection of 10% chloral hydrate solution at the single dose of 0.3ml per 100g body weight. The rat was fixed in supine position with four legs stretched outward. The abdominal skin was prepped by hair shaving followed by disinfection with 70% alcohol. A 5 cm long midline incision was made to open the abdominal cavity. The small bowel and colon were pulled out of the abdominal cavity and left right to the abdomen beyond the field of irradiation. The small bowel and colon were placed on a piece of gauze that was wetted with warm saline to keep them moist. The rat was transferred to the chamber of RS2000 pro biological irradiator (RADSOURCE, USA). After the chamber door was securely closed, the rat was irradiated in a ventrodorsal direction with 160 kV X-ray operating at 25 mA and filtered with 0.3 mm of copper. The total irradiation dosage was 35 Gy which was delivered at the rate of 1.75 Gy/min. The irradiation was localized to a square-shaped field of 3 cm × 3 cm encompassing the central abdominal region by using a lead shield (Figure 2). The viscera especially small bowel and colon were left off the irradiation field to avoid the devastating gastrointestinal adverse effect. After completion of irradiation, the rat was taken out. In RT-only model, the rat was not subjected to aortic transplantation. The small bowel and colon were pushed back to the abdominal cavity, and the abdominal incision was closed by 3-0 Vicryl suture. The rat was kept in warming blanket until recovery from anesthesia. However, in RT-plus-TX model, abdominal aorta was procured immediately from the irradiated rat and transplanted to a healthy counterpart.

### 2.3. Aorta Transplantation

#### 2.3.1. Instruments and Reagents

The surgical instruments and reagents are listed as follows: JSZ6 stereo microscope (Jiangnan Novel Optics, China), a package of rodent surgical instruments including a needle holder, a pair of scissors, two pairs of forceps and a Colibri retractor (RWD Life Science, China), a set of microsurgical instruments which consisted of a microneedle holder, two microclamps, a pair of microscissors and two pairs of microtweezers (Jinzhong Medical Instrument, China), 9-0 nylon suture with 1/2 circle taper point needle (Jinhuan Medical Products, China), 3-0 coated Vicryl suture with 3/8 circle taper point needle (Ethicon, USA), a 2 *μ*l pipette with tips (Eppendorf, Germany), Histoacryl blue tissue adhesive (B.Braun, Germany), 10% chloral hydrate solution (Leagene Biotechnology, China), normal saline (Baxter, China), and heparin (Qianhong Bio-pharm, China). All surgical instruments are sterilized before use.

#### 2.3.2. Surgical Procedures

The procedures of rat aorta transplantation were described in previous studies with minor modifications [17]. The rat was fixed on the operating table after anesthesia with 10% chloral hydrate solution. The small bowel and colon were pushed aside to expose abdominal aorta by using a Colibri retractor. The infrarenal aorta was carefully dissected away from adjacent tissue. Lumbar arteries branching from aorta were ligated with 9-0 nylon suture. A 1.5 cm long aortic graft was procured after blood flow of aorta was blocked by ligation right below infrarenal artery and at aortic bifurcation. The aortic graft was perfused with 125 u/ml heparin solution to wash the vessel clear of all blood components and then stored at 4°C. The donor rat was euthanized by cervical dislocation. The recipient was a healthy F344 rat. The procedures of anesthesia, skin prepping, and separation of abdominal aorta referred to the donor operation. Blood flow of aorta was temporarily blocked by inserting two microclamps: one right below renal branch and the other at aortic bifurcation. The abdominal aorta was transected at the midpoint of renal arteries and aortic bifurcation. The cut ends were rinsed with heparin solution. The graft aorta was anastomosed to the recipient aorta in an end-to-end manner by running stitches with 9-0 nylon suture. After the completion of anastomosis, the microclamps were removed to restore blood flow. Two methods were recommended if anastomotic bleeding occurred. First, simply press the anastomosis with a dry cotton swab for 30 sec if bleeding was not serious. Otherwise, use the microclamps again to stop bleeding and then apply a 0.5 ul aliquot of Histoacryl blue tissue adhesive along the anastomotic line by a 2 ul pipette [18]. It usually took less than 5 sec to form a strong and transparent layer of hemostat covering around the anastomosis. Then the microclamps were taken away to check the patency and bleeding of anastomosis. Of note, applying too much adhesive would result in anastomotic stenosis and subsequent thrombus formation. The abdominal incision was closed by running suture with 3-0 Vicryl. The recipient was kept in warming blanket until recovery from anesthesia.

#### 2.3.3. Postoperative Treatment

The recipient was fed on water and normal diet. On postoperative day 90, the recipient was euthanized to procure the graft aorta for biomedical analysis. The vascular anastomoses were carefully removed to avoid the effect of suture material on evaluation of vasculopathy.

### 2.4. MSC Infusion

Bone marrow MSCs of male F344 rats (Cyagen Biosciences, China) were cultured in Dulbecco's modified Eagle's medium (DMEM; Gibco, Thermo Fisher Scientific) supplemented with 10% fetal bovine serum (FBS; Gibco, Thermo Fisher Scientific, USA) at 37°C with a humidified atmosphere of 5% CO2 and 95% air. The MSCs were labelled with green fluorescence protein (GFP) by viral transfection as previously reported [19]. The rats were infused with GFP-labelled MSCs via tail vein at the dose of 2 × 10^6^ cells × 4 times starting from the 30th day after irradiation or operation with the interval of 15 days. The GFP-labelled MSCs were freshly prepared in serum-free medium before infusion, and cell infusion was performed after the rat was anesthetized with 10% chloral hydrate solution.

### 2.5. Histology Analysis

The specimens of aorta were fixed with 10% formalin, paraffin-embedded, and cross-sectioned at 5 *μ*m intervals. The sections were stained with hematoxylin-eosin and Masson's trichrome to evaluate vasculopathy. The expression of myeloperoxidase (MPO) in tissue section was analyzed by using standard avidin-biotin complex technique [20]. The antibodies were purchased from Agilent Technologies, China, and listed as follows: polyclonal MPO antibody (catalog A0398), biotinylated anti-rabbit secondary antibody (catalog E0353), and horseradish peroxidase-conjugated streptavidin (catalog P0397). Intimal thickness was normalized to full thickness of vascular wall to obtain the relative value.

### 2.6. Fluorescent Staining

Fresh graft aorta was mounted in OCT compound and cut into 5 *μ*m cross sections. The nuclei were stained with 4′,6-diamidino-2-phenylindole (DAPI). The GFP-labelled cells were counted in the sections under a fluorescent microscope. The average number of GFP-labelled cells per high power field (HPF) was calculated from three random HPFs for each rat and eight rats for each group.

### 2.7. Real-Time Quantitative Reverse Transcription Polymerase Chain Reaction (PCR)

#### 2.7.1. Total RNA Extraction

Fresh graft aorta tissue was stored in nitrogen immediately after it was harvested. A total of 40 mg aortic sample was collected from eight rats of the same group with 5 mg from each rat. Aortic tissue was homogenized by Dounce tissue grinder in ice bath and dissolved in 1 ml Trizol solution (Catalog R0016, Beyotime, China). The homogenate was transferred into an Eppendorf tube and centrifuged at 12,000×g for 10 min at 4°C. The supernatant was transferred to a prechilled fresh Eppendorf tube and kept at room temperature for 5 min before 0.2ml chloroform was added. The mixture was vortexed for 15 sec and left at room temperature for 3 min followed by centrifugation at 12,000×g for 15 min at 4°C. Aqueous phase (the top phase) was transferred to a fresh tube and mixed with 0.5ml isopropanol. The mixture was incubated at room temperature for 10 min before centrifugation at 12,000×g for 10 min at 4°C. The supernatant was disposed. The RNA pellet was washed with 1ml of 75% alcohol followed by centrifugation at 7,500×g for 5 min at 4°C. The supernatant was removed, and the pellet was air-dried for 3 min. The RNA pellet was dissolved in 20*μ*l of DEPC water and stored at -70°C.

#### 2.7.2. Reverse Transcription

A 10*μ*l aliquot of reaction mixture was prepared for each reaction. The microtube was mounted with 2 *μ*l of 5×PrimeScript RT Master Mix (catalog RR036, Takara, China), 2 *μ*l of 100 ng/*μ*l total RNA solution, and 6 *μ*l of ultrapure water as required to reach 10 *μ*l. Reverse transcription was performed in Applied Biosystem 7500 by using the program setting: 15 min at 37°C followed by 5 sec at 85°C.

#### 2.7.3. PCR Analysis for cDNA Samples

A 20*μ*l aliquot of reaction mixture was prepared for each reaction. The microtube was mounted with 10 *μ*l of 2×SYBR Premix Ex Taq II (catalog RR820L, Takara, China), 0.8 *μ*l of forward primer, 0.8 *μ*l of reverse primer, 0.4 *μ*l of 50×ROX Reference Dye, 2 *μ*l of cDNA solution, and 6 *μ*l of ultrapure water as required to reach 20 *μ*l. The primers were prepared by Sangon biotech, China, according to the reported sequence [21, 22] as follows: tumor necrosis factor *α* (TNF*α*) forward primer 5'-CACGCTCTTCTGTCTACTGA-3' and reverse primer 5'-GGACTCCGTGATGTCTAAGT-3', transforming growth factor *β* (TGF*β*) forward primer 5'-CCTGGGCACCATCCATGA-3' and reverse primer 5'-CAGGTGTTGAGCCCTTTCCA-3', interleukin 1*β* (IL-1*β*) forward primer 5'-GGGTTGAATCTATACCTGTCCTGTGT-3' and reverse primer 5'-GACAAACCGCTTTTCCATCTTCT-3', interleukin 2 (IL-2) forward primer 5'-CAGCTCGCATCCTGTGTTGCAC-3' and reverse primer 3'-GCTTTGACAGATGGCTATCCATC-3'. The PCR was performed in Applied Biosystem 7500 by using the program setting: 30 sec at 95°C followed by 40 cycles of 5 sec at 95°C and 30 sec at 60°C. The target RNA expression of each group was normalized to that of *β*-actin and vehicle control by using the comparative cycle threshold method.

### 2.8. Real-Time Quantitative PCR

#### 2.8.1. Genomic DNA Preparation

Total DNA was extracted by using genomic DNA mini preparation kit with spin column (Beyotime, China). Briefly, a fresh Eppendorf tube was mounted with 10 mg aortic sample from each group. The sample was incubated in the mixture of 180 *μ*l lysis buffer A and 20 *μ*l proteinase K solution at 55°C for 3 hr to allow it to dissolve completely. Then 200 *μ*l lysis buffer B was added and incubated at 70°C for 10 min. After 200 *μ*l anhydrous ethanol was added, the mixture was loaded to DNA purification column and centrifuged at 6,000 × g for 1 min. The column was washed with 500 *μ*l buffer I and centrifuged at 6,000 × g for 1 min followed by being washed with 600 *μ*l buffer II and centrifuge at 18,000 × g for 2 min. Finally, 200 *μ*l the column was loaded with elution buffer, kept still at room temperature for 3 min, and centrifuged at 18,000 × g for 1 min. A fresh Eppendorf tube was placed under the column to collect all elution which contained purified DNA.

#### 2.8.2. PCR Analysis for DNA Samples

A 20 *μ*l aliquot of reaction mixture was prepared for each reaction. The microtube was mounted with 10 *μ*l of 2×SYBR Green qPCR Master Mix (catalog 638320, Takara, China), 1 *μ*l of forward primer, 1 *μ*l of reverse primer, 2 *μ*l of 50 ng/*μ*l DNA template, and 6 *μ*l of ultrapure water as required to reach 20 *μ*l. The primers for sex determination region on the Y chromosome (Sry) and for the housekeeping gene *β*-actin was prepared according to the reported sequence [23]: Sry forward primer 5'-GAGGCACAAGTTGGCTCAACA-3' and reverse primer 5'-CTCCTGCAAAAAGGGCCTTT-3', *β*-actin forward primer 5'-CCATTGAACACGGCATTG-3' and reverse primer 5'-TACGACCAGAGGCATACA-3' (Sangon biotech, China). The PCR was performed in Roche LightCycler system by using the program setting: 30 sec at 95°C followed by 40 cycles of 5 sec at 95°C and 30 sec at 60°C. The Sry DNA level was normalized to that of *β*-actin by using the comparative cycle threshold method.

### 2.9. Statistical Analysis

Statistical analysis was performed using GraphPad Prism 5. Data were expressed as mean± standard deviation. Group comparison was made by using Mann–Whitney* U* test. A P value <0.05 was considered to indicate statistical significance.

## 3. Results and Discussion

**Figure 3:**
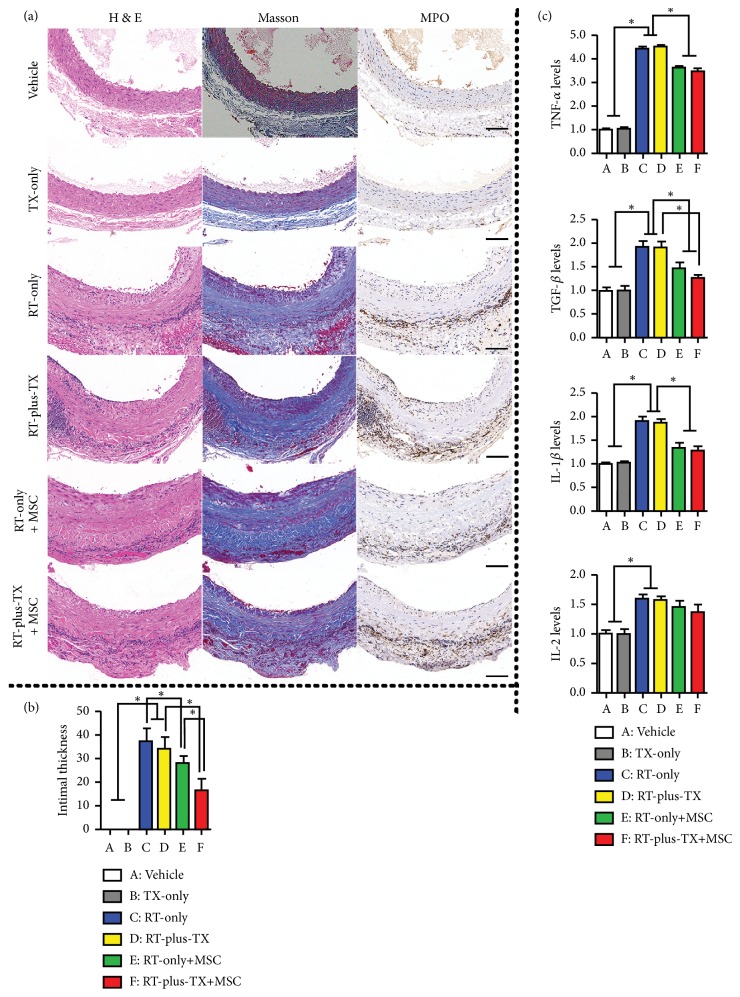
Histology and cytokine analysis of abdominal aortas. (a) Cross-sectional images of abdominal aorta. Serial cross sections of aorta were processed with hematoxylin-eosin stain (H&E), Masson's trichrome stain (Masson), and immunostaining with antimyeloperoxidase antibody using DAB substrate kit (MPO), respectively. The images represented the investigation of eight rats for each group. Scar bar 100 *μ*m. (b) Histological analysis of intimal hyperplasia. The relative intimal thickness was normalized to full thickness of vascular wall and expressed as a percentage. Eight rats were investigated for each group. Group comparison was performed with Mann–Whitney* U* test. ^*∗*^*P* < 0.05. (c) Expression of proinflammatory cytokines in aortas. The cytokine levels were measured by real-time qualitative reverse transcription PCR and normalized to vehicle control. The experiment was repeated three times for each group. Group comparison was performed with Mann–Whitney* U* test. ^*∗*^*P* < 0.05.

The MSC-based therapy holds great promise for radiation injury. Many animal experiments have demonstrated that transplantation of MSCs attenuated radiation injury by inhibiting inflammatory response and promoting tissue regeneration [12, 24, 25]. The homing of MSCs to injured tissue is the prerequisite for generating the effect [8, 14, 15]. However, only a few studies clearly have shown how many cells finally migrate and engraft into irradiated tissues [23, 26]. Moreover, highly sensitive methods like PCR analysis for specific biomarkers of transplanted MSCs are preferred to quantify the MSC recruitment in many researches [16, 23, 26]. This suggests a very low population of homed MSCs. Although the poor engraftment of MSCs would be multifactorial, the animal model was intrinsically relevant. Despite the use of a beam limiting device to avoid unnecessary tissue irradiation, the body area that was eventually irradiated not only included abdominal aorta but also adjacent tissue, both of which were likely to release damage signal to stimulate MSC migration [27–30]. Thus, circulating MSCs would also be distributed into adjacent tissue, which was unintentionally subjected to radiation injury yet forming a larger compartment of MSC homing than irradiated aorta. In that case, the frequency of MSCs being recruited to irradiated aorta would be greatly decreased. Conversely, if the aorta was exclusively subjected to radiation injury, more circulating MSCs would gather in irradiated aorta. The theory was supported by an early study which found that local irradiation promoted the migration of MSCs to the irradiated field [16]. Therefore, this study introduced the RT-plus-TX model. In this model, the aorta of irradiated rat was anatomically separated from adjacent tissue and transplanted to the healthy counterpart. On postoperative day 90, the segment of irradiated graft aorta was procured for histological analysis. The vascular injury consisted of intimal hyperplasia and vascular fibrosis (Figure 3), which resembled the histological changes of irradiated vessels in humans [3, 5]. The hyperplastic intima was formed by accumulation of abundant spindle-shaped cells and extracellular matrix mixed with some degree of inflammatory cell infiltration. In Masson's trichrome stain, the amount of blue-stained collagen fiber was increased in all layers of irradiated aorta, suggesting diffuse vascular fibrosis after irradiation. Moreover, a large number of MPO-positive cell gathered in the adventitia of irradiated aorta. This indicated that severe oxidative stress occurred as it was commonly present in radiation injury [31]. This study also designed three control groups for RT-plus-TX group in order to validate the impact of irradiation on the vascular injury and rule out the possible interference from aorta transplantation (Figure 1). The RT-only group was used as negative control for aorta transplantation, and the TX-only and vehicle groups served as negative control for irradiation. In comparison, the histological response was almost the same between the RT-plus-TX and RT-only groups while the aorta remained almost normal in the TX-only and vehicle groups. Next, the homogenate of irradiated aorta was sent to PCR analysis of inflammatory cytokines, among which TNF-*α*, TGF-*β*, IL-1*β*, and IL-2 were selected to have significant relevance to radiation-induced vascular injury [32–34]. All cytokines were significantly increased in RT-plus-TX group as compared to the TX-only and vehicle control groups (Figure 3). The cytokine levels were comparable between RT-plus-TX and RT-only groups. This suggested proinflammatory response to radiation injury in the RT-plus-TX group, which was consistent with previous studies [31, 34]. Altogether, the RT-plus-TX model showed typical features of radiation-induced vascular injury.

**Figure 4:**
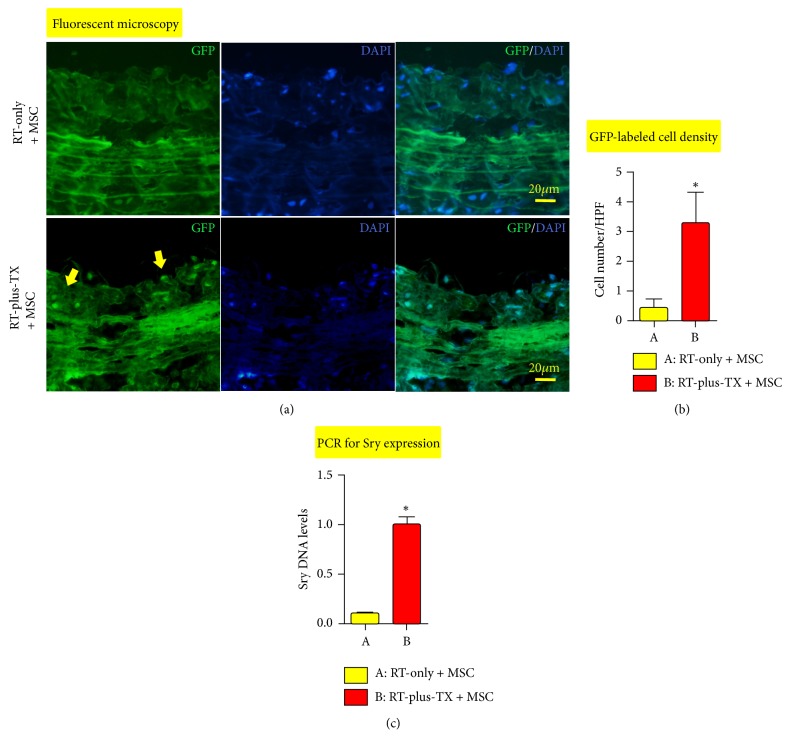
Engraftment of mesenchymal stem cells (MSC) in irradiated aorta. MSC engraftment was investigated by two techniques: fluorescent microscopy for tracing cells with green fluorescent protein (GFP) label and PCR analysis for sex determination region on the Y chromosome (Sry) specifically carried by transplanted MSCs. For each group, eight rats were investigated by fluorescent microscopy, and the PCR analysis of aortic tissue homogenates from eight rats was repeated three times. Group comparison was performed with Mann–Whitney* U* test. ^*∗*^*P* < 0.05.

Then we investigated whether the frequency of MSC engraftment was increased in this model as expected. The MSCs were infused to the RT-plus-TX rats starting from 30 days after irradiation, repeating for four times with the interval of 15 days. In this study, the transplanted MSCs were isogenic to host rats, by which immune rejection was avoided to increase cell survival in vivo. The MSCs were obtained from male rats, and thus they can be traced by Sry gene after infusion to female host rats. We also labelled the MSCs with GFP to render them visible under a fluorescent microscope. Fifteen days after the last MSC infusion (ninety days after aorta irradiation), the graft aortas were processed to the sections stained with DAPI. As a result, the GFP-labelled cells were preferably engrafted into intima layer at the average density of 3.30 cells/HPF in the RT-plus-TX + MSC group. In contrast, GFP-labelled cells were nearly invisible in the RT-only + MSC group. The result was supported by PCR analysis for the Sry gene. The RT-plus-TX + MSC group had a significantly higher level of Sry gene than the RT-only + MSC group (Figure 4). Moreover, the high frequency of MSC engraftment was correlated with the significant relief of vascular injury in RT-plus-TX + MSC group when compared to vascular injury in RT-only + MSC group. Of note, the intimal thickness was significantly lower in RT-plus-TX + MSC group than RT-only + MSC group. The declining TGF-*β* level of irradiated aortas in RT-plus-TX + MSC group probably suggested the inhibition of fibrosis in vascular remodeling given that TGF-*β* signaling plays a critical role in vascular fibrosis [35] (Figure 3). Taken together, RT-plus-TX model promoted the engraftment of MSC into irradiated aortas yielding a relevant benefit on vascular injury.

To better understand why RT-plus-TX model improved MSC engraftment, we preformed the following calculations.

In the RT-only model, the volume of irradiated tissue (V_RT-only_) was calculated by multiplying length (L), width (W), and depth (D) of body compartment exposed to radiation. The length and width were determined by the square-shaped irradiation field of 3cm × 3cm, and the depth was the thickness of rat posterior abdominal wall, approximately 1.5cm on average (Figure 2). The result was shown as follows:

(1) VRT-only=3  cm  L×3  cm  W×1.5  cm  D=13.5  cm3

In the RT-plus-TX model, aortic graft was the only tissue with irradiation. The graft was shaped like a cylinder with a height (H) of 1.5 cm and a cycle base area of 1 mm in diameter (A) (Figure 2). The formula to calculate the irradiated volume (V_RT-plus-TX_) was shown as follows:

(2) VRT-plus-TX=1.5  cm  H×π×0.1cmA22=0.012  cm3

If the migrating MSCs were evenly distributed in the irradiated tissue, and if the number of migrating MSCs was constant (i.e., the sum of infused MSCs possessing the high capacity of migration in vivo was not different between the RT-only + MSC and RT-plus-TX + MSC groups), then the MSC density of irradiated tissue was inversely proportional to the volume of irradiated tissue. Therefore, the MSC density (DEN) was calculated from the following formula:

(3) DENRT-plus-TXDENRT-only=VRT-onlyVRT-plus-TX=13.5  cm30.0118  cm3=1.14×103

**Figure 5:**
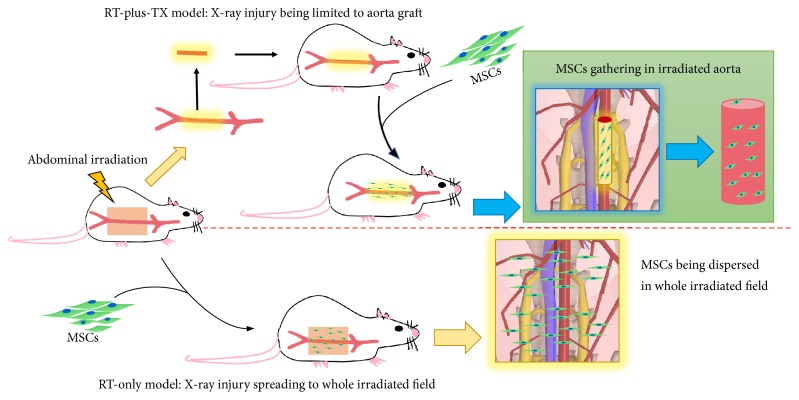
Illustrative mechanism by which RT-plus-TX model promotes the engraftment of mesenchymal stem cells (MSCs). After local irradiation, the aortas together with adjacent tissues are subjected to injury and release damage signal to attract the migration of infused MSCs. The migrated MSCs are dispersed in the aortas and adjacent tissues, leading to seemly low frequency of MSC engraftment. When the irradiated aortas are transplanted to isogenic healthy rats, the injured aortas live with normal surrounding tissues. The MSCs are prone to gather in the injured aortas, and the frequency of MSC engraftment is improved.

The irradiated aorta of RT-plus-TX+ MSC group was estimated to have approximately one-thousand-fold higher MSC density than that of RT-only+ MSC group. In that case, the density of GFP-labelled cells in the RT-only + MSC group was anticipated to be 0.0029 (3.30 / 1.14 × 10^3^) cells/HPF which suggested a fairly low incidence of detecting GFP-labelled cells under fluorescent microscopy. The estimate was supportive of what was observed in this experiment, although many factors like whether the migrating MSCs were evenly distributed between the aorta and adjacent tissue were neglected. Intriguingly, the previous study revealed that MSCs were preferably homed to the viscera, skin, and muscle after local irradiation [16]. The MSC engraftment to aortas was not suggested if the authors did not ignore the possibility and checked the aortas. In other words, the aorta might not be the favorable destination for MSCs as compared to adjacent tissue and organs if the same dose irradiation was given. Therefore, the sequestration of irradiated aorta was presumably helpful to diminish the preference of MSC recruitment to adjacent tissue. Generally, the RT-plus-Tx model supported the theory that simultaneously irradiated adjacent tissue interfered with the gathering of MSCs to irradiated aorta. The sequestration of irradiated rat aorta by transplantation to a healthy counterpart was an effective way to improve MSC local recruitment (Figure 5).

However, the RT-plus-TX model had some drawbacks. First, this model was suitable for the study of large vessels but not microvascular system. Radiation vasculitis was morphologically different depending on the size of vessels. When compared with large vessels, capillary vessels were prone to rupture, dilate, and form thrombus after irradiation [5]. Such pathological features were not present in the RT-plus-TX model. Moreover, the method to sequester irradiated vessels as described in this model was not applicable for microvasculature, since transplantation of capillary vessels alone was technically difficult. Second, the RT-plus-TX model was not easy-to-use especially for beginners who had no training for microsurgery. The major obstacle was to complete aortic anastomosis in a short time without serious complication like bleeding and thrombosis. Therefore, the technique of cyanoacrylate-assisted vascular anastomosis was introduced to simplify the procedure of aorta transplantation and improve successful rate. The use of cyanoacrylate was safe and effective enough as reported in many studies [18, 36, 37]. Third, some might argue that the RT-plus-TX model was not reliable because surgical trauma such as ischemia reperfusion injury would accelerate progression of radiation vasculitis. Admittedly, surgical trauma was unavoidable in this model, given that aorta transplantation was the indispensable step to sequester irradiated aorta. But most follow-up effect of surgical trauma was temporary, being initiated shortly after operation and regressing within one month according to the previous study [18]. Moreover, the interference of surgical trauma was well controlled by setting the RT-only group which served as sham surgery control. Consequently, the RT-plus-TX group shared similar pathological features of radiation vasculitis as the RT-only group. We also ruled out the possibility that the early surgical trauma would promote the late MSC engraftment in this study. The MSCs were infused to the TX-only rats at the same dose, but there were no MSCs in aorta grafts (Figure S1). Last, the contribution of immune rejection was eliminated by transplantation between F344 inbred rats which possessed minimal genetic difference within the strain.

In conclusion, the RT-plus-TX model promotes MSC accumulation in irradiated vessels by separating irradiated vascular segment from adjacent tissue. This model is preferred in the study of MSC-based therapy for radiation vasculitis when the evaluation of MSC homing is demanding.

## Data Availability

Data are available upon request to authors.

## Supplementary Materials

Supplementary Materials Figure S1: Fluorescent microscopy view of the aortas from the TX-only rats after infusion of GFP-labelled mesenchymal stem cells.
