# Supplementary material for: A Rat Model of Radiation Vasculitis for the Study of Mesenchymal Stem Cell-Based Therapy
Source: Biomed Res Int. 2019 Mar 6;2019:3727635. doi: 10.1155/2019/3727635 (PMC6431386; doi:10.1155/2019/3727635)

Figure S1: Fluorescent microscopy view of the aortas from the TX-only rats after infusion of GFP-labelled mesenchymal stem cells
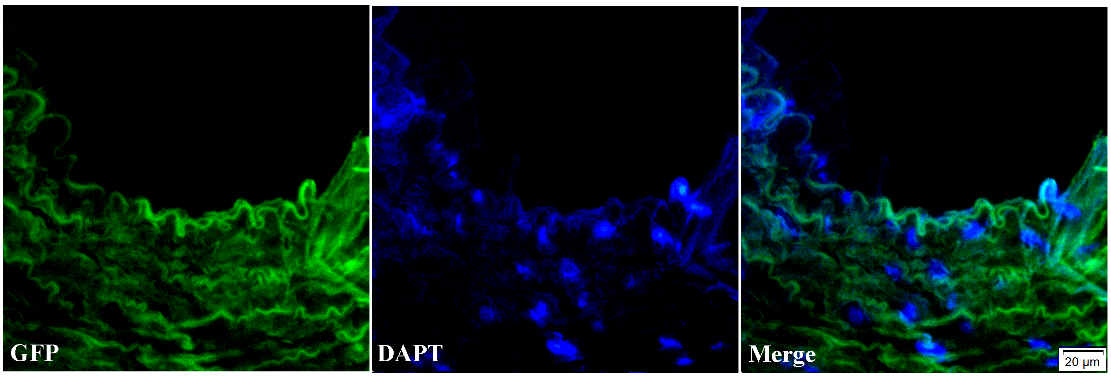

Supplement: Supplementary Materials — Figure S1: Fluorescent microscopy view of the aortas from the TX-only rats after infusion of GFP-labelled mesenchymal stem cells. [file 3727635.f1.docx]
